# It takes two to tango: two TatA paralogues and two redox enzyme-specific chaperones are involved in the localization of twin-arginine translocase substrates in *Campylobacter jejuni*

**DOI:** 10.1099/mic.0.080713-0

**Published:** 2014-09

**Authors:** Yang-Wei Liu, Andrew Hitchcock, Robert C. Salmon, David J. Kelly

**Affiliations:** Department of Molecular Biology and Biotechnology, University of Sheffield, Firth Court, Western Bank, Sheffield S10 2TN, UK

## Abstract

The food-borne zoonotic pathogen *Campylobacter jejuni* has complex electron transport chains required for growth in the host, many of which contain cofactored periplasmic enzymes localized by the twin-arginine translocase (TAT). We report here the identification of two paralogues of the TatA translocase component in *C. jejuni* strain NCTC 11168, encoded by *cj1176c* (*tatA1*) and *cj0786* (*tatA2*). Deletion mutants constructed in either or both of the *tatA1* and *tatA2* genes displayed distinct growth and enzyme activity phenotypes. For sulphite oxidase (SorAB), the multi-copper oxidase (CueO) and alkaline phosphatase (PhoX), complete dependency on TatA1 for correct periplasmic activity was observed. However, the activities of nitrate reductase (NapA), formate dehydrogenase (FdhA) and trimethylamine N-oxide reductase (TorA) were significantly reduced in the *tatA2* mutant. In contrast, the specific rate of fumarate reduction catalysed by the flavoprotein subunit of the methyl menaquinone fumarate reductase (MfrA) was similar in periplasmic fractions of both the *tatA1* and the *tatA2* mutants and only the deletion of both genes abolished activity. Nevertheless, unprocessed MfrA accumulated in the periplasm of the *tatA1* (but not *tatA2*) mutant, indicating aberrant signal peptide cleavage. Surprisingly, TatA2 lacks two conserved residues (Gln8 and Phe39) known to be essential in *Escherichia coli* TatA and we suggest it is unable to function correctly in the absence of TatA1. Finally, only two TAT chaperones (FdhM and NapD) are encoded in strain NCTC 11168, which mutant studies confirmed are highly specific for formate dehydrogenase and nitrate reductase assembly, respectively. Thus, other TAT substrates must use general chaperones in their biogenesis.

## Introduction

*Campylobacter jejuni* is a Gram-negative epsilonproteobacterium that is one of the most frequent causes of bacterial gastroenteritis worldwide and which has a major impact on both public health and economic activity (Jacobs-Reitsma *et al.*, 2008). The bacterium is present at high levels as an intestinal commensal in many bird species and some animal species; human consumption of contaminated poultry meat is the most common source of infection (Wagenaar *et al.*, 2008). Despite the importance of *C. jejuni* as a pathogen, far less is known about its fundamental physiology and metabolism compared with other enteric pathogens (for a recent review, see Stahl *et al.*, 2012). One of the key phenotypic features of *C. jejuni* is its microaerophilic nature. Growth of most strains is inhibited under fully aerobic conditions, which in part is due to the utilization of essential oxygen-sensitive enzymes in central metabolic pathways (Kendall *et al*., 2014). Nevertheless, *C. jejuni* is a respiratory bacterium and has a complex branched electron transport system that facilitates both oxygen-dependent and oxygen-independent energy conservation and growth under varying environmental conditions (Kelly, 2008).

The principal pathways of electron transport in *C. jejuni* have been elucidated by a combination of bioinformatic and experimental approaches, which has revealed several novel features (Guccione *et al.*, 2010; Pittman *et al.*, 2007; Sellars *et al.*, 2002; Smart *et al.*, 2009), although there are still many electron transport genes of unknown function. A variety of inorganic and organic compounds, including hydrogen, sulphite, thiosulphate, formate, lactate and gluconate, can be utilized as electron donors (Liu *et al.*, 2013; Myers and Kelly, 2005; Pajaniappan *et al.*, 2008; Thomas *et al*., 2011; Weerakoon *et al.*, 2009). Electrons can be transferred to two terminal oxidases, either directly from the menaquinone pool to a quinol oxidase (CioAB) or via the proton-translocating cytochrome *bc_1_* complex and cytochrome *c* to the *cb*-oxidase (Jackson *et al.*, 2007). Several diverse types of terminal reductase that allow alternative electron acceptors to oxygen to be utilized are also present. As extreme oxygen limitation will be encountered in the intestine, some of these enzymes are thought to be important for persistence of *C. jejuni* in the mucosa while others may be involved in detoxification reactions. Examples include two distinct fumarate reductases, Frd and Mfr (Guccione *et al.*, 2010; Weingarten *et al.*, 2009), nitrate and nitrite reductases (Pittman *et al.*, 2007), trimethylamine N-oxide (TMAO)/DMSO reductase (Sellars *et al.*, 2002; Weingarten *et al.*, 2008), two cytochrome *c* peroxidases (Bingham-Ramos & Hendrixson, 2008) and a novel bi-functional cytochrome *c* tetrathionate reductase (Liu *et al.*, 2013).

Many of these electron transport enzymes are localized in the periplasm. Gram-negative bacteria utilize two pathways for transporting proteins destined for the periplasm across the cytoplasmic membrane: the general secretion (Sec) pathway and the twin-arginine translocase (TAT) pathway. While the Sec system translocates unfolded proteins using energy derived from both ATP hydrolysis and the proton motive force, the TAT pathway transports pre-folded proteins across the cytoplasmic membrane (for a recent review see Patel *et al*., 2014). TAT translocation is independent of ATP hydrolysis and is energized solely by the membrane potential (Yahr & Wickner, 2001). Substrates of the TAT pathway include enzymes with cytoplasmically inserted cofactors (Berks, 1996), multi-subunit complexes that require assembly prior to export (Rodrigue *et al.*, 1999) and cofactorless proteins whose folding is incompatible with the Sec system (Rose *et al.*, 2002). The TAT system is also involved in integration of a subset of inner membrane proteins (Hatzixanthis *et al.*, 2003) and outer membrane biosynthesis (Stanley *et al.*, 2001).

Our current understanding of the mechanism of TAT-mediated transport in Gram-negative bacteria has largely been elucidated in the model organism *Escherichia coli*. Substrate proteins bearing a signal peptide, which has the highly conserved twin-arginine motif S/T-R-R-x-F-L-K (where x is a polar amino acid), at the n/h-region boundary (Berks, 1996) are targeted to an oligomeric membrane-integrated translocase complex consisting of three proteins, TatA, TatB and TatC (Bogsch *et al.*, 1998; Sargent *et al.*, 1998, 1999). TatA and TatB are homologous, each containing a single N-terminal transmembrane anchor, a short-hinged region, an amphipathic helix and a charged C-terminal region (Hicks *et al.*, 2003), while TatC is a polytopic membrane protein with six transmembrane domains (Behrendt *et al.*, 2004). Despite their similarity, TatA and TatB carry out unique roles and cannot normally functionally substitute for one another (Sargent *et al.*, 1999). TatB and TatC form a stable complex that recognizes and binds the signal peptide of TAT substrates (Bolhuis *et al.*, 2001; Tarry *et al.*, 2009). It is thought that TatC recognizes the twin-arginine motif (Alami *et al.*, 2003) and that TatB forms an oligomeric binding site for folded TAT precursors (Maurer *et al.*, 2010). Recent evidence from studies of the chloroplast TAT system (Aldridge *et al.*, 2014) suggests that TatC subunits are arranged in a concave face-to-face arrangement, creating a closed chamber. The substrate signal peptide seems to insert into spaces between the TatC subunits, forcing them apart and allowing access of TatA to the chamber, which is proposed to seed TatA polymerization and translocase assembly (Aldridge *et al.*, 2014), but the actual mechanism by which the substrate protein is translocated by TatA is incompletely understood. Originally, it was thought to be exported through an aqueous pore formed by the TatA monomers (Gohlke *et al.*, 2005), the number of which governs the pore diameter. However, recent structural studies favour a model in which TatA polymerization thins and disorders the membrane to produce transient rupture (Rodriguez *et al.*, 2013).

TatD and E proteins are present in some organisms. TatD is a soluble cytoplasmic protein with DNase activity but is not a component of the TAT pathway (Lindenstrauss *et al.*, 2010; Wexler *et al.*, 2000). The *tatE* gene is a shorter paralogue of *tatA* thought to have arisen from a cryptic gene duplication; TatE is at least partially functionally interchangeable with TatA but appears to be largely redundant (Sargent *et al.*, 1998). Note that some Gram-positive bacteria may differ in their *tat* gene complement and can contain several independent TatA–TatC systems (Goosens *et al.*, 2014).

In *C. jejuni*, alkaline phosphatase (Cj0145) and the nitrate reductase NapA subunit were the first proteins shown to be TAT substrates (in strain 81116; van Mourik *et al.*, 2008). In our previous study (Hitchcock *et al.*, 2010), proteomics and activity measurements with an isogenic *tatC* mutant and a complemented strain were used to experimentally verify the TAT dependence of NapA and the majority of the other proteins that are predicted to be exported via the TAT pathway in strain NCTC 11168. A study of the TAT system in *C. jejuni* 81-176 (Rajashekara *et al.*, 2009) showed that a *tatC* null mutant was more sensitive to antimicrobials, and defective in biofilm formation, motility, flagellation, survival under osmotic shock, and oxidative and nutrient stress, although many of these phenotypes are likely to be due to indirect effects of the mutation.

In this paper, we report the identification of two unlinked *tatA* genes in *C. jejuni* and an investigation of their roles by the analysis of selected TAT substrate enzyme activities and periplasmic localization in single and double *tatA* mutants and complemented strains. We found that deletion of *tatA1* (*cj1176c*) resulted in a much larger effect on growth and enzyme localization than deletion of *tatA2* (*cj0786*). Although the *tatA2* gene is encoded within the periplasmic nitrate reductase (*nap*) operon, *tatA1* deletion abolished NapA activity in intact cells and periplasmic fractions while *tatA2* deletion reduced it by only ~50 %, suggesting another role for TatA2. We observed that TatA2 can substitute for TatA1 in the assembly of the unidirectional periplasmic-facing methyl menaquinone fumarate reductase, MfrA, but with aberrant signal peptide cleavage. Overall, our mutant studies suggest that TatA2 is not completely functional in the absence of TatA1. Directly upstream of *tatA2* in the same operon is *cj0785* encoding a TAT chaperone or redox enzyme maturation protein (REMP; Turner *et al.*, 2004) homologous to NapD in *E. coli* and other bacteria. REMPs are small cytoplasmic proteins that bind tightly to TAT signal peptides and which serve to co-ordinate cofactor insertion with translocation through the TAT system, thus preventing premature export (Dow *et al.*, 2014; Jack *et al.*, 2004). Despite using many different cofactor-containing TAT-dependent electron transport enzymes, *C. jejuni* appears to possess only one other REMP, namely FdhM, a TorD homologue encoded by *cj1514c* upstream of the formate dehydrogenase (*fdh*) operon, which we have shown is only required for Fdh activity (Hitchcock *et al.*, 2010). This raises questions about the specificity of NapD. Here, we show that NapD is specifically involved in NapA assembly.

## Methods

### 

#### Bacterial strains, media and culture conditions.

*C. jejuni* strain NCTC 11168 was routinely cultured at 37 °C under microaerobic conditions [10 % (v/v) O_2_, 5 % (v/v) CO_2_ and 85 % (v/v) N_2_] in a MACS-VA500 growth cabinet (Don Whitley Scientific) on Columbia agar containing 5 % (v/v) lysed horse blood and 10 µg ml^−1^ each of amphotericin B and vancomycin. To select *C. jejuni* mutants, kanamycin or chloramphenicol was added at a final concentration of 30 µg ml^−1^. Liquid cultures of *C. jejuni* were routinely grown in brain heart infusion (BHI) broth or Mueller–Hinton broth (Oxoid) supplemented with 20 mM l-serine (MH-S) under standard microaerobic conditions (gas concentrations as above), with 50–100 ml of medium contained in 250 ml conical flasks with continuous orbital shaking at 180 r.p.m. For oxygen-limited cultures, the diffusion of oxygen was severely restricted by using 500 ml medium contained in a 500 ml conical flask without shaking, as described previously (Liu *et al.*, 2013, Sellars *et al.*, 2002). Electron acceptors were added from filter-sterilized stock solutions to a final concentration of 20 mM. Growth curves shown are from representative single experiments but all growth experiments were repeated at least three times with similar results. For induction of alkaline phosphatase in *C. jejuni* a method modified from that of van Mourik *et al.* (2008) was used. Phosphate-free Dulbecco’s modified Eagle's medium (DMEM; Gibco) with 20 mM l-serine, 20 mM HEPES buffer, pH 7, and appropriate antibiotics was the basal medium. Cells were grown microaerobically with an initial concentration of 1.6 mM [Pi] and then incubated with 0.08 mM [Pi] in the same basal medium for induction of alkaline phosphatase. *E. coli* DH5α was cultured in Luria–Bertani (LB) broth or agar at 37 °C. Carbenicillin (50 µg ml^−1^), kanamycin (50 µg ml^−1^) or chloramphenicol (30 µg ml^−1^) was added where indicated.

#### DNA isolation, manipulation and construction of mutants.

Standard techniques were employed for the transformation preparation, and restriction analysis of plasmid DNA from *E. coli* (Sambrook *et al.*, 1989). Phusion proofreading DNA polymerase (Thermo Scientific) was used routinely for PCR, and the primers used are detailed in Table 1. The isothermal assembly (ISA) cloning method (Gibson *et al.*, 2009) was employed to generate plasmid constructs for the mutagenesis of *cj1176c* (*tatA1*) and *cj0786* (*tatA2*). The DNA fragments to be assembled in the ISA reaction were prepared as follows. The vector pGEM3Zf(-) was digested with *Hin*cII and phosphatase treated. For *cj1176c*, primer pairs pGEM-1176-5F/Kan-1176-5R and Kan-1176-3F/pGEM-1176-3R (30 bp adaptor plus 20 bp *cj1176c* sequence) were designed to amplify two PCR products: fragment 1 (F1; 5′ end of the *cj1176c* gene plus upstream flanking DNA) and fragment 2 (F2; 3′ end of the *cj1176c* gene plus downstream flanking DNA). A similar strategy was followed for *cj0786*, using the analogous primer pairs shown in Table 1. The adaptor sequences used when amplifying F1 and F2 were designed such that the adjacent DNA fragments to be joined share single stranded terminal sequence overlaps with the vector and with a kanamycin (for *cj1176c*) or a chloramphenicol (for *cj0786*) resistance cassette, derived from pJMK30 or pAV35 (van Vliet *et al.*, 1998), which were separately PCR amplified using primers Kan-F and Kan-R or Cat-F and Cat-R (Table 1). ISA reactions were purified using the QIAquick PCR purification kit, eluting in 15 µl distilled H_2_O. The resulting DNA (5 µl) was used to transform competent *E. coli* DH5α, with selection on LB agar containing kanamycin or chloramphenicol. Colonies were screened by PCR with the flanking primers (pGEM-1176-5F/pGEM-1176-3R and pGEM-0786-5F/pGEM-0786-3R). Correct constructs designated pM*tatA1* and pM*tatA2* were selected and the insert sequence was confirmed by automated DNA sequencing using M13 primers (Core Genomic Facility, University of Sheffield Medical School, UK). Plasmids were introduced into *C. jejuni* NCTC 11168 by electroporation and transformants were selected using Columbia blood agar plates supplemented with kanamycin or chloramphenicol. A *tatA1/tatA2* double mutant was constructed by introducing pM*tatA2* into the *tatA1* mutant. Mutants were confirmed by colony PCR with the primers detailed above. To generate a *napD* (*cj0785*) insertion mutant, primers napD-F and napD-R were used to amplify a 763 bp product including the coding region of *napD* (339 bp) and 200 bp flanking DNA. The PCR product was digested and cloned into *Bam*HI-digested pGEM3Zf(-) and transformants were selected as above. The *cat* cassette from pAV35 was blunt end cloned into the unique *Swa*I site in *napD* in the same transcriptional orientation to generate plasmid p*napDcat*. A construct for deletion of *napD* was generated using a modified overlap extension PCR method (Wurch *et al.*, 1998). Primers napD-del1 and napD-del2 were used to generate a 636 bp fragment consisting of 18 bp at the beginning of *napD* plus ~580 bp upstream DNA. Primers napD-del3 and napD-del4 were used to generate a 626 bp fragment consisting of 12 bp at the end of *napD* plus ~560 bp downstream DNA. These fragments contained 20 bp of complementary sequence at their extreme 3′ and 5′ ends, respectively, such that when mixed they would anneal and primers napD-del1 and napD-del4 could be used to generate a 1242 bp PCR product comprising only the very beginning and ends of *napD* (plus flanking DNA), with a *Xho*I site introduced at the junction. This fragment was cloned into the *Bam*HI site of pGEM3Zf(-) and then the *cat* cassette blunt end cloned into the engineered *Xho*I site in the same transcriptional orientation (confirmed by sequencing), producing the plasmid p*napDdel*. Plasmids p*napDcat* and p*napDdel* were introduced into strain NCTC 11168 by electroporation as described above with selection on chloramphenicol.

**Table 1.** Primers used in this study

Name	Sequence (5′ to 3′)
pGEM-1176-5F	GAGCTCGGTACCCGGGGATCCTCTAGAGTCTTTAGAATGGGCTAGAGTGC
Kan-1176-5R	AAGCTGTCAAACATGAGAACCAAGGAGAATACCAACCACCCATTTTATTC
Kan-1176-3F	GAATTGTTTTAGTACCTAGCCAAGGTGTGCGCTTAAGGTTTAGTCTTTTG
pGEM-1176-3R	AGAATACTCAAGCTTGCATGCCTGCAGGTCTACCCGCATCATTGACATAG
pGEM-0786-5F	GAGCTCGGTACCCGGGGATCCTCTAGAGTCCACAAGTGATTTTAAGCCTG
CAT-0786-5R	AAGCTGTCAAACATGAGAACCAAGGAGAATCATATACTTTACACTTTAAG
CAT-0786-3F	GAATTGTTTTAGTACCTAGCCAAGGTGTGCAAAACAAAATAGGCATTAAA
pGEM-0786-3R	AGAATACTCAAGCTTGCATGCCTGCAGGTCTTTTATCTTCTAAGTCTTGC
Kan-F	ATTCTCCTTGGTTCTCATGTTTGACAGCTTAT
Kan-R	GCACACCTTGGCTAGGTACTAAAACAATTCAT
ISACAT-F	ATTCTCCTTGGTTCTCATGTTTGACAGCTTGAATTCCTGCAGCCCGGGGG
ISACAT-R	GCACACCTTGGCTAGGTACTAAAACAATTCACTAGTGGATCCCGGGTACC
napD-F	TAATGA***GGATCC***TTGTGCTTTAAGTCCTAG
napD-R	TGCAGT***GGATCC***ATGATTTTTAATGCCTATTTT
CAT-F	ATTCTCCTTGGGAATTCCTGCAGCCC
CAT-R	GCACACCTTGGACTAGTGGATCCCGG
napD-del1	TGACGCTA***GGATCC***AAAGCTCGAAGAAAATAGCAT
napD-del2	ATGCGTA***CTCGAG***GGTACTTACTAGAAAGATTATTCATCAATAC
napD-del3	AAGTACC***CTCGAG***TACGCATCAATTTTCTTAAAGTGTAAAGTATATG
napD-del4	TGACTGTA***GGATCC***TGTCTTCTTTGCTCTTCCATG
1176comp-F	GAAGG**CGTCTCACATG**GGTGGTTGGTCAAGTCCAAG
1176comp-R	TCAA**CGTCTCACATG**TTAAGCTTTTTTTGTTTCGTCTATACTTG
napDcomp-F	ATGCAT**CGTCTCACATG**AATAATCTTTCTAGT
napDcomp-R	ATGCAT**CGTCTCACATG**TTAAGAAAATTGATT
786comp-F	CTTAAAGTG**CGTCTCACATG**GTATTTTTAATCCCATTGC
786comp-R	TATG**CGTCTCACATG**CTATTTTGTTTTAAACAATTTTTC
cj0046-F	GAGCCAATCCTATTTCATCAGCTATG
1176semi-F	GAATGAAGGAGAATAAAATGG
1176semi-R	CTTAAGCTTTTTTTGTTTCGTC
0786semi-F	AAAATGAAAATGCTGAGAATG
0786semi-R	TGATTTTTAATGCCTATTTTG
gyrAsemi-F	GTTATTATAGGTCGTGCTTTG
gyrAsemi-R	CAAAGTTGCCTTGTCCTGTAA

*Esp*3I sites are underlined in bold; *Bam*HI (GGATCC) and *Xho*I (CTCGAG) sites are given in bold italics.

#### Construction of complemented strains.

The complete ORFs of *cj1176c* (*tatA1*), *cj0785* (*napD*) and *cj0786* (*tatA2*) were PCR amplified respectively using the primer pairs 1176comp-F/1176comp-R, napDcomp-F/napDcomp-R and 786comp-F/786comp-R (Table 1). The resulting fragments were *Esp*3I digested and cloned into the *BsmB*I site of the pC*metK* (for *tatA1*) or pK*metK* (for *tatA2* and *napD*) vectors (Gaskin *et al.*, 2007) to create plasmids pC*metA1*, pK*metA2* and pK*napD*. They were introduced into the appropriate mutant background, with selection for chloramphenicol or kanamycin resistance. These complementation plasmids integrate the intact wild-type gene driven by the constitutive *metK* promoter at the *cj0046c* pseudogene locus, which was confirmed by colony PCR using gene-specific primers and the cj0046-F primer (Table 1).

#### Reverse transcription (RT)-PCR.

Overnight cultures of wild-type NCTC 11168, *tatA1* and *tatA2* mutants grown in MH-S media were harvested and resuspended in 1 ml TRI reagent (Sigma) and RNA isolation procedures were carried out according to the manufacturer’s instructions. Total RNA preparations were DNase treated using the Turbo DNA-free kit (Ambion). The RNA concentration and purity was determined using a BioPhotometer (Eppendorf) and all RNA samples were stored at −80 °C. For cDNA synthesis, 5 µg total RNA was used in 20 µl reverse transcription reactions (SuperScript III Reverse Transcriptase; Invitrogen) according to the manufacturer’s instructions. Synthesized cDNA (2 µl) was mixed with 2× Mytaq (Bioline), 5 nM forward and reverse primers (Table 1) for *cj1176c*, *cj0786* or *gyrA* to a final volume of 50 µl. The PCR mastermix was aliquoted to six tubes, which were removed at cycle numbers 10, 15, 20, 25, 30 and 35 during the PCRs and were separated on 1.3 % agarose gels.

#### Cellular fractionation.

For the preparation of cell-free extracts, 50 ml cultures were harvested at 10 000 ***g*** for 15 min at 4 °C. The pellet was resuspended in 2.5 ml 25 mM phosphate buffer, pH 7.4, and cell-free extracts were prepared by sonication, as described by Velayudhan & Kelly (2002). For the preparation of *C. jejuni* periplasm, a cold osmotic shock method developed previously (Liu *et al.*, 2013; Myers & Kelly, 2005) was employed. Contamination of the periplasm by cytoplasmic proteins was controlled for by immunoblotting with *E. coli* anti-GroEL antibody (Sigma), which cross-reacts with *C. jejuni* GroEL.

#### Immunoblotting.

Immunoblotting was carried out according to Huang *et al.* (2007) and Guccione *et al.* (2010) with slight modifications: cell-free extracts or periplasmic samples were denatured by boiling in Laemmli sample buffer and separated by SDS-PAGE on 8 % (w/v) acrylamide gels and electroblotted onto nitrocellulose membrane (Hybond-C extra; GE Healthcare). Blocking was carried out with Gelatin-NET at room temperature for 1 h. A buffer containing 0.2 % (w/v) BSA and 2 % (w/v) polyvinylpyrolidone (PVP; Mw 24 000– 40 000) in PBS/Tween-20 (PBST) was used for membrane washing and in the dilution of anti-MfrA (Guccione *et al.*, 2010), anti-GroEL and horseradish peroxidase (HRP)-conjugated secondary antibodies (Sigma). The HRP-conjugated bound antibodies were detected using the enhanced chemiluminesence (ECL) kit from GE Healthcare.

#### Enzyme assays.

Viologen-linked assays were performed under strictly anaerobic conditions at 37 °C using a Shimadzu UV-2401PC spectrophotometer as previously described (Hitchcock *et al.*, 2010). Sulphite oxidase (and in some cases formate dehydrogenase) was measured by substrate-dependent oxygen uptake assays, as previously described (Myers & Kelly, 2005; Thomas *et al.*, 2011). Phenoloxidase assays for CueO were performed with periplasm as described previously (Hall *et al.*, 2008). The alkaline phosphatase assay was modified from that of van Mourik *et al.* (2008). The OD_600_ of overnight DMEM cultures with 0.08 mM [Pi] was measured and the cells were harvested and resuspended in 0.9 ml 1 M Tris/HCl, pH 8.5. Then 0.1 ml of 10.75 mM *p*-nitrophenyl phosphate (Sigma) was added and the reaction was maintained at 37 °C for 5–10 min. To stop phosphatase activity, the reaction tube was chilled on ice, then 0.1 ml ice-cold 1 M K_2_HPO_4_ was added and the cells were microfuged at 13 000 ***g*** for 5 min. The supernatant was collected and the *A*_550_ and *A*_420_ were measured. The units of alkaline phosphatase were calculated by the formula 10^3^×[*A*_420_ − (1.75×*A*_550_)]/*t*×OD_600_×*V* where *t* is incubation time (minutes) and V is cell volume (ml). Protein concentrations of cell suspensions or periplasm were determined using the method of Markwell *et al.* (1978) or the dye-binding Bio-Rad assay, respectively.

## Results

### Identification, occurrence, mutagenesis and expression analysis of two *tatA* genes in *C. jejuni*

**Fig. 1.**
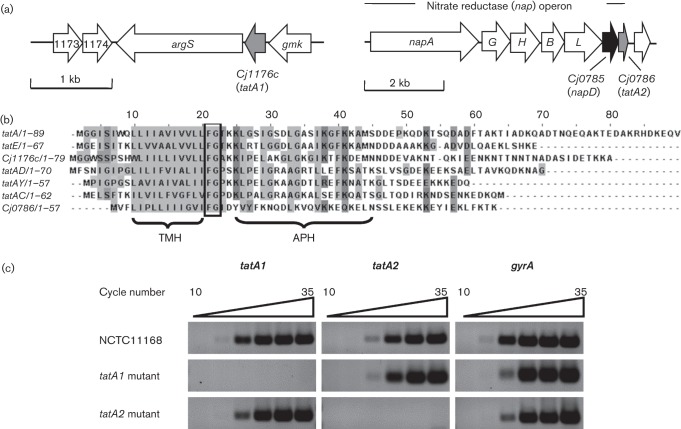
The identification of *tatA1* (*cj1176c*) and *tatA2* (*cj0786*) in *C. jejuni* strain NCTC 11168. (a) *tatA1* (*cj1176c*) is located between *gmk* (guanylate kinase) and *argS* (arginyl-tRNA synthetase) genes whereas *tatA2* (*cj0786*) is part of the *nap* (nitrate reductase) operon, immediately downstream of a *napD* homologue (*cj0785*). Mutants in *tatA1*, *tatA2* and *napD* were made as described in Methods. (b) Sequence alignment of TatA/E proteins. TatA and TatE are from *E. coli* strain MG1655. TatAY, TatAD and TatAC are from *Bacillus subtilis* strain 168. The boxed, highly conserved, region is the ‘FG’ hinge between the transmembrane helix (TMH) and amphipathic helix (APH). (c) RT-PCR of *tatA1*, *tatA2* and *gyrA* (control housekeeping gene) expression in *C. jejuni* NCTC 11168, *tatA1* and *tatA2* mutant strains. Primers used for gene-specific amplification are listed in Table 1 (1176semiF to gyrAsemiR). Agarose gels of amplicons resulting after the number of PCR cycles indicated are shown.

TatA/E proteins have an N-out, C-in topology and are characterized by the possession of a hydrophobic N-terminal transmembrane helix, followed by a hinge region with a highly conserved FG sequence and then an amphipathic helix associated with the inner surface of the cytoplasmic membrane (Koch *et al.*, 2012; Porcelli *et al.*, 2002). The *cj1176c* gene in *C. jejuni* NCTC 11168 encodes a TatA homologue that has all of these features (Fig. 1a, b), although no functional studies of this gene have been reported. However, sequence comparisons and secondary structure predictions of the product of the *cj0786* gene showed that it also encoded a protein with the appropriately placed helices and the conserved FG hinge region (Fig. 1b), although it is shorter than Cj1176 (57 compared with 79 aa) due to truncation at the C terminus. It is thus more like the *E. coli* TatE protein. We designated *cj1176* as *tatA1* and *cj0786* as *tatA2.* A bioinformatic analysis of epsilonproteobacterial genome sequences showed that *tatA1* genes were present in all species examined, whereas *tatA2* was absent in many species and, of those strains examined, only present in the related group of *C. jejuni*, *C. coli*, *C. lari* and *C. upsaliensis* (Table S1 and Fig. S1, available in the online Supplementary Material). In each case, the *tatA2* genes are located at the distal end of *nap* operons. In *C. lari* and *C. upsaliensis*, the TatA2 proteins lack the conserved glycine of the FG hinge region (Fig. S1).

Using *C. jejuni* NCTC 11168, we constructed individual deletion mutants of *tatA1* and *tatA2*, complemented strains where the respective wild-type gene was integrated at the *cj0046c* pseudogene locus (*tatA1*^+/−^ and *tatA2*^+/−^) and also a double *tatA1tatA2* mutant (see Methods). RT-PCRs carried out with the wild-type, *tatA1* and *tatA2* strains (Fig. 1c) showed that both *tatA* genes were expressed in wild-type cells and that deletion of either *tatA* homologue did not appear to significantly affect expression of the other.

### Growth phenotypes of the *tatA* mutants under aerobic and oxygen-limited conditions

**Fig. 2.**
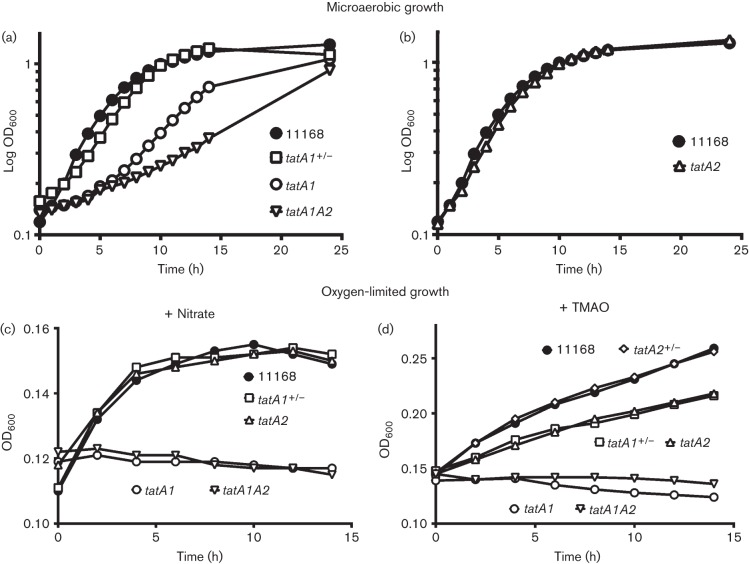
Microaerobic (a, b) and oxygen-limited (c, d) growth curves of wild-type, *tatA* mutants and complemented strains. For microaerobic growth, 100 ml volumes of MH-S medium in 250 ml conical flasks were shaken at 180 r.p.m. in a gas atmosphere of 10 % (v/v) oxygen, 5 % (v/v) carbon dioxide and 85 % (v/v) nitrogen. For oxygen-limited growth, cultures were incubated at 37 °C in almost completely filled 500 ml unshaken conical flasks containing BHI medium supplemented with 20 mM nitrate (c) or 20 mM TMAO (d) as electron acceptors. None of the strains grew without any electron acceptors under oxygen limitation (data not shown). The data shown are representative of at least three independent growth experiments.

Fig. 2 shows the growth characteristics of the wild-type, mutants and complemented strains under standard microaerobic growth conditions and under oxygen-limited conditions with either nitrate or TMAO as electron acceptors. Deletion of *tatA1* had a severe effect on oxygen-dependent growth, which was fully reversed in the complemented strain (Fig. 2a), while deletion of *tatA2* did not noticeably affect the growth rate or final cell density (Fig. 2b). However, the double *tatA1tatA2* strain reproducibly grew less well with oxygen than the *tatA1* mutant (Fig. 2a), suggesting a role for TatA2, at least in the absence of TatA1.

The periplasmic molybdoenzymes nitrate reductase and TMAO reductase have previously been shown to be TatC dependent (Hitchcock *et al.*, 2010; van Mourik *et al.*, 2008) and are the sole reductases for nitrate and TMAO, respectively, in strain NCTC 11168. Given the position of the *tatA2* gene within the *nap* gene cluster, we were particularly interested to determine if TatA2 was a Nap-specific TAT component. In the presence of nitrate, mutation of *tatA1* completely abolished oxygen-limited growth and complementation with the *tatA1* gene restored growth to wild-type levels (Fig. 2c). However, the *tatA2* mutant grew as well as the wild-type with nitrate as electron acceptor under these conditions (Fig. 2c). In the presence of TMAO, the *tatA1* mutant also showed no growth, but the *tatA2* mutant displayed a slightly lower growth rate than the wild-type, which was restored by complementation (Fig. 2d). For the double *tatA1A2* mutant, no growth was observed with either nitrate or TMAO (Fig. 2c, d). None of the strains grew without any exogenous electron acceptors under oxygen limitation (data not shown). Thus, these data suggest that TatA2 is not required for the assembly of the periplasmic nitrate reductase, but that it may have some role in the biogenesis of the TMAO reductase.

### Dependence of the assembly of cofactor-containing electron transport enzymes on TatA1 and TatA2

**Fig. 3.**
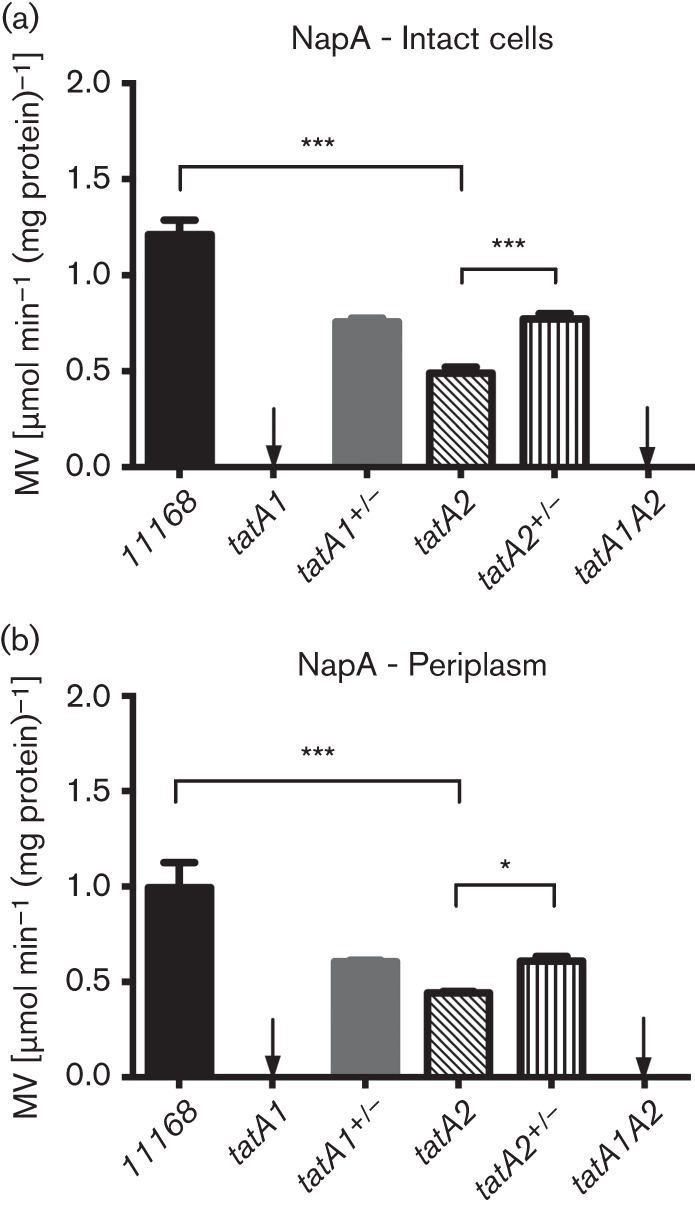
Comparison of nitrate reductase activities in (a) intact cells and (b) periplasmic fractions of wild-type, *tatA* mutants and complemented strains. The nitrate-dependent oxidation of reduced methyl viologen (MV) was measured as described in Methods. Relevant significant differences in activity are indicated by asterisks (***P<0.001, *P<0.05) according to Student’s *t*-test. The data shown are means±sd of three independent experiments.

As a measure of the correct export of the TAT-dependent nitrate reductase subunit NapA to the periplasm we compared nitrate-dependent reduced methyl viologen oxidation rates in intact cells and periplasmic extracts of the wild-type, *tatA* mutants and complemented strains (Fig. 3). Methyl viologen does not readily cross the inner membrane and in all cases the pattern of activities observed was highly similar in both intact cells (Fig. 3a) and the corresponding periplasmic fractions (Fig. 3b). Mutation of *tatA1* alone resulted in the total abolition of nitrate reductase activity, while mutation of *tatA2* reduced the rate significantly, to ~ 50 % of the wild-type rate. The complemented strains showed a partial restoration of activity, probably due to lower than optimal gene expression from the *metK* promoter. These data show that TatA1 has the major role in the export of NapA, consistent with the growth data above, but also suggests that TatA2 might play a minor role.

**Fig. 4.**
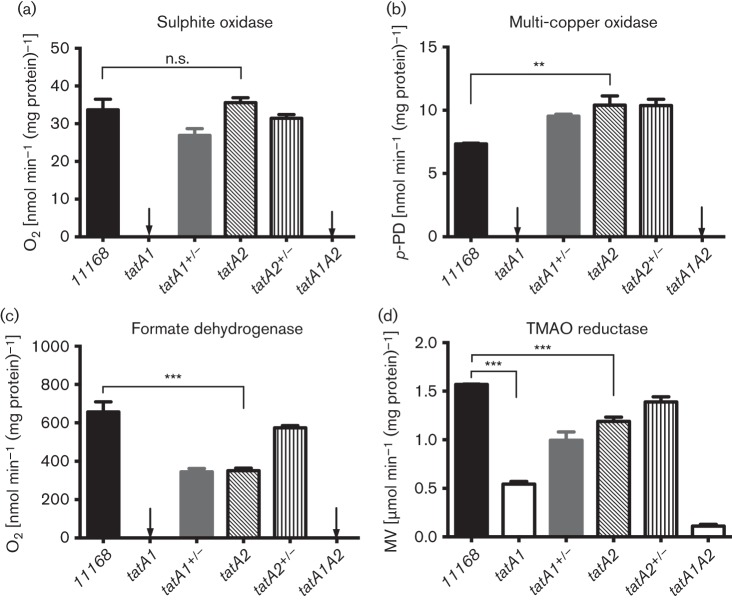
The activities of key TAT-dependent electron transport enzymes in intact cells of wild-type, mutant and complemented strains. Assay methods were as described in Methods. Relevant significant differences in activity are indicated by asterisks (***P<0.001, **P<0.01) according to Student’s *t*-test (n.s.; no significant difference). The data shown are means±sd of at least three independent experiments. *p*-NP, *p*-nitrophenyl phosphate; MV, methyl viologen.

An examination of the TatA1 or TatA2 dependency of the activity of several other TAT-dependent cofactor-containing electron transport enzymes in intact cells (TMAO reductase, formate dehydrogenase and sulphite oxidase) or periplasmic fractions (the multi-copper oxidase CueO/Cj1516) is shown in Fig. 4. For sulphite oxidase (Fig. 4a), a complete dependency on TatA1 was evidenced by undetectable sulphite respiration in intact cells of the *tatA1* and double mutant, but no significant difference between activities in the wild-type and *tatA2* strain. A very similar pattern was seen for the multi-copper oxidase CueO, with the *tatA2* mutant showing even slightly higher activity than the wild-type (Fig. 4b). In contrast, a partial dependency on TatA2 was observed for formate dehydrogenase (Fig. 4c), although, as with NapA, mutation of *tatA1* alone resulted in undetectable enzyme activity. With TMAO reductase (Fig. 4d), a significant activity remained in the *tatA1* mutant, indicating continued export of TorA to the periplasm. This could be attributed to TatA2 by the pattern of activities in the *tatA2* and double mutant, the latter exhibiting just a very low residual rate.

### Export of the periplasmic fumarate reductase subunit MfrA occurs via either TatA1 or TatA2

**Fig. 5.**
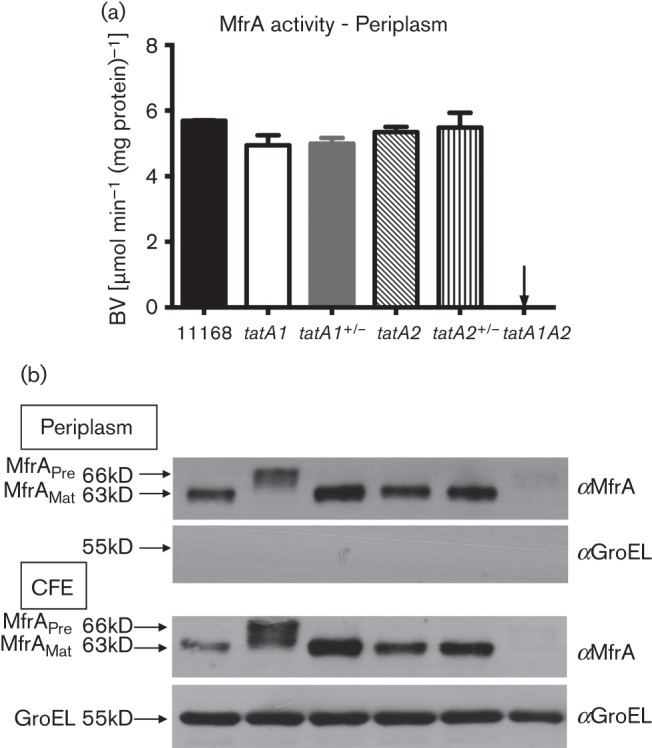
Dual dependence of MfrA translocation on TatA1 and TatA2. (a) Activity of MfrA in periplasmic extracts of the strains shown (means±sd of three independent experiments), as measured by the fumarate-dependent oxidation of reduced benzyl viologen (BV). (b) Corresponding immunoblots of both periplasmic fractions (upper two panels) and cell-free extracts (CFE; lower two panels), probed with either anti-MfrA or anti-GroEL antibodies. The latter was used as a control for cytoplasmic contamination of the periplasm. Approximately 5 µg periplasmic protein and 15 µg CFE protein was loaded in each lane.

Unusually, *C. jejuni* possesses two fumarate reductases, one acting as a reversible bi-functional succinate dehydrogenase/fumarate reductase (Frd) that is cytoplasmic-facing and non-TAT dependent, while the other (methylmenaquinone fumarate reductase, Mfr) is a periplasmic-facing enzyme that acts as a unidirectional fumarate reductase (Guccione *et al.*, 2010; Weingarten *et al.*, 2009). The active site subunit of the latter enzyme, MfrA, has a twin-arginine signal sequence and its export was shown to be unequivocally TAT dependent in studies with a *tatC* mutant (Hitchcock *et al.*, 2010). Here, we investigated which TatA paralogue was required for MfrA translocation to the periplasm by measuring fumarate-dependent reduced benzyl viologen oxidation in periplasmic extracts and by immunoblotting with anti-MfrA antibodies. Fig. 5(a) shows that in marked contrast to the other electron transport enzymes studied above, inactivation of either *tatA1* or *tatA2* individually had no effect on MfrA activity in the periplasm. However, this activity was abolished in the *tatA1 tatA2* double mutant. The corresponding immunoblots of the periplasmic fractions (Fig. 5b) show that the MfrA subunit is indeed translocated in both the single *tatA1* and *tatA2* mutants, while it is absent in the double mutant. However, a higher molecular mass form (~66 kDa) corresponding to the size expected of the unprocessed protein is clearly present in the *tatA1* periplasm, accompanied by a smear suggesting some degradation, while in the *tatA2* mutant periplasm only the ~63 kDa band corresponding to the mature form is visible, suggesting normal processing. Complementation with the wild-type *tatA1* gene restored normal processing in the *tatA1* mutant. A similar pattern was seen in total cell-free extracts, but in the double mutant there was no evidence of the accumulation of the unprocessed MfrA, indicating that it is degraded in this mutant background (Fig. 5b). Together, these data suggest redundancy between TatA1 and TatA2 for the translocation of MfrA, but also reveal an unexpected processing defect when the cells are forced to use TatA2 in the *tatA1* mutant background.

### Tat-dependent but cofactorless enzyme alkaline phosphatase (PhoX) is translocated exclusively via TatA1

**Fig. 6.**
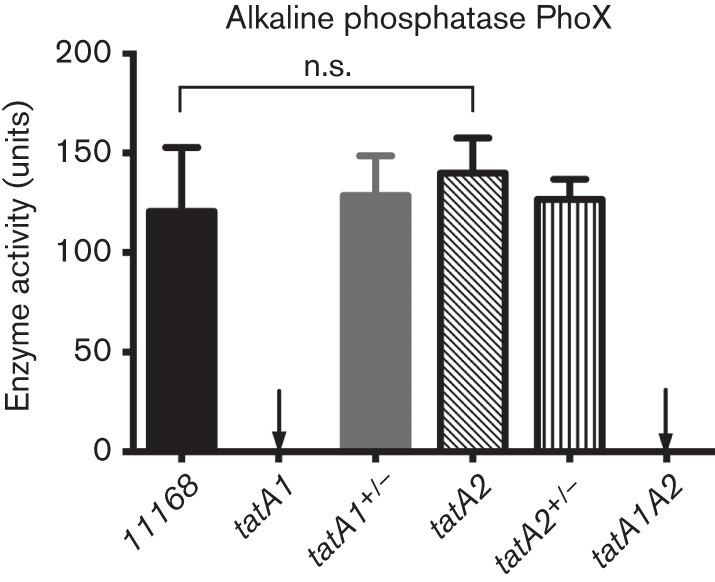
The non-cofactor-containing enzyme alkaline phosphatase (PhoX) is exclusively translocated via TatA1. Rates of hydrolysis of *p*-nitrophenyl phosphate were measured in phosphate-limited intact cells without lysis using the assay described in Methods. The units of activity are normalized to cell density (see van Mourik *et al.*, 2008 and Methods). The data shown are means±sd of three independent experiments (n.s., no significant difference).

The TAT-dependent enzymes studied above are all involved in electron transport reactions in the periplasm of *C. jejuni*, and possess complex cofactors, which explain their requirement for transport through the TAT system. Of the TAT substrate proteins in *C. jejuni*, the alkaline phosphatase encoded by *cj0145* (PhoX; van Mourik *et al.*, 2008) is unique in being a hydrolytic enzyme that requires calcium ions but otherwise has no known cofactor. PhoX may be an example of a TAT substrate that has folding requirements or kinetics that are incompatible with Sec translocation (van Mourik *et al.*, 2008). Fig. 6 shows the results of assays for alkaline phosphatase activity in intact cells of wild-type, mutant and complemented strains grown under conditions of phosphate limitation to induce *phoX* expression. Deletion of *tatA1* completely abolished PhoX activity while complementation with the wild-type *tatA*1 gene completely restored it. In contrast, deletion of *tatA2* did not affect PhoX activity. The data thus indicate a complete dependence of PhoX translocation on TatA1.

### NapD is a Nap-specific REMP but only complete deletion of *napD* abolishes nitrate reductase activity and nitrate-dependent growth

**Fig. 7.**
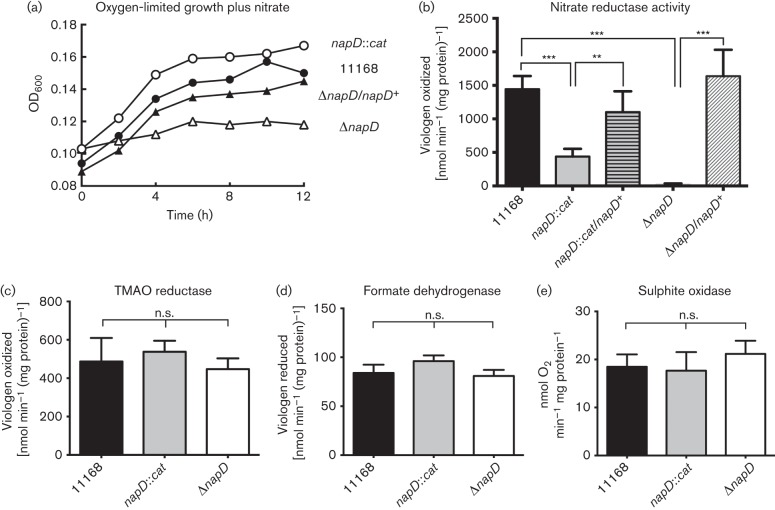
Effect of insertional inactivation or complete deletion of the putative *C. jejuni* NapA chaperone gene *napD*. (a) Cells of the wild-type (filled circles), *napD* : : *cat* (open circles), the deletion strain Δ*napD* (open triangles) and the complemented strain Δ*napD/napD^+^* (filled triangles) were grown under oxygen-limited conditions in BHI medium plus 20 mM sodium nitrate. (b–e) Activities of the redox enzymes shown were assayed in intact cells of the wild-type, mutants and complemented strains, as described in Methods. Relevant significant differences in activity are indicated by asterisks (***P<0.001, **P<0.01) according to Student’s *t*-test (n.s.; no significant difference). The data shown are means±sd of at least three independent experiments.

**Fig. 8.**
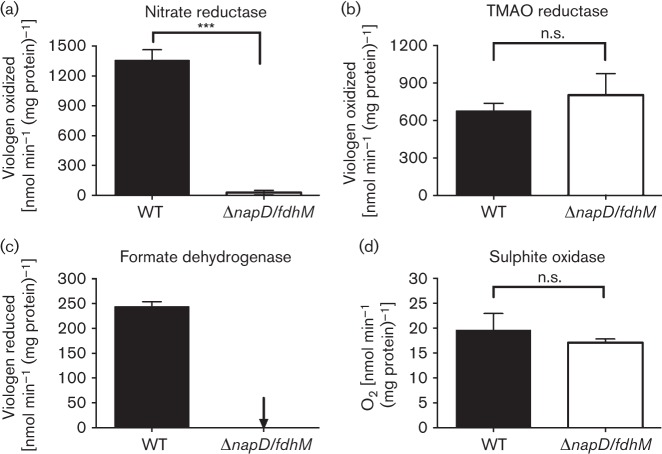
Activity of TAT-dependent redox enzymes in a Δ*napD/fdhM* double mutant. Cells were grown for ~16 h under microaerobic conditions in MH-S media and were harvested and assayed as described in Methods. Nitrate reductase (a) and TMAO reductase (b) activities were measured spectrophotometrically using reduced methyl viologen. Formate dehydrogenase (c) and sulphite oxidase (d) were assayed spectrophotometrically by reduction of oxidized methyl viologen and by substrate-dependent oxygen uptake, respectively. The specific activities shown are means±sd of three independent experiments. Relevant significant differences in activity are indicated by asterisks (***P<0.001) according to Student’s *t*-test (n.s., no significant difference).

The gene immediately upstream of *tatA2* in the *nap* operon (*cj0785*) is a homologue of *napD*. Cj0785 is thus predicted to be an REMP for the nitrate reductase in *C. jejuni* as is the case for NapD in *E. coli* (Potter & Cole, 1999). It would be expected that inactivation of *napD* would prevent oxygen-limited growth with nitrate. This was found to be the case for the Δ*napD* mutant, where no significant nitrate-dependent growth was observed (Fig. 7a). Complementation of the Δ*napD* strain with the wild-type *napD* gene restored growth (Fig. 7a). However, a *napD* : : *cat* insertion mutant was able to grow as well as the wild-type when supplied with nitrate (Fig. 7a). The Δ*napD* strain was completely devoid of nitrate reductase activity when assayed using reduced methyl viologen, but the *napD* insertion mutant retained approximately 30 % of the activity of the wild-type parent, thus explaining the growth phenotype (Fig. 7b). Complementation with the wild-type copy of *napD* in strains *napD : : cat/napD^+^* and Δ*napD/napD^+^* restored nitrate reductase activity to approximately wild-type levels in each case (Fig. 7b), confirming these phenotypes are a direct result of the different *napD* mutations. Importantly, the activities of TMAO reductase, formate dehydrogenase and sulphite oxidase were not affected by mutation of *napD* in either the insertion or the deletion strain (Fig. 7c–e). The only other REMP encoded in *C. jejuni* NCTC 11168 is FdhM (Cj1514), a TorD homologue known to be required for maturation of formate dehydrogenase (Hitchcock *et al.*, 2010). A Δ*napD fdhM* double mutant displayed no formate dehydrogenase or nitrate reductase activity, but sulphite oxidase and TMAO reductase activities were unaffected (Fig. 8). This additive phenotype indicates complete specificity in these two REMPs for their single enzyme clients.

## Discussion

Our identification of two *tatA* homologues in the genome of *C. jejuni* is especially interesting in the context of the relative complexity of the electron transport systems in this bacterium, despite its small genome size and evident host adaptation (Kelly, 2008). In fact, rather few Gram-negative bacteria have been identified that have duplicated *tat* genes. As in *C jejuni*, the commonest situation is the presence of an additional TatA homologue, which has variably been called TatE or TatA2. In *E. coli*, *tatE* is expressed at low levels and deletion of *tatE* has no effect on TAT transport or cell viability (Jack *et al.*, 2001). TatE is C-terminally truncated compared with TatA, but functional studies show that, like TatA, TatE can translocate substrates of varying size (Baglieri *et al.*, 2012). Interestingly, in biofilms cells express the *tatE* gene at a higher level than in planktonic cells (Beloin *et al.*, 2004), hinting at a specialized role under stress conditions. In contrast, in the denitrifying bacterium *Pseudomonas stuzeri* a *tatE* gene is located in the *nos* gene cluster, required for nitrous oxide reduction, and was shown to be essential for denitrification (Heikkilä *et al.*, 2001), presumably because it is specifically required for the translocation of the NosA polypetide to the periplasm. In the Gram-negative predatory bacterium *Bdellovibrio bacteriovorus*, *tatA1* and *tatA2* genes have also been identified and here TatA2 was shown to be essential for both host-dependent and host-independent growth, while deletion of *tatA1* slowed the rates of growth in each mode (Chang *et al.*, 2011). The TAT system in this bacterium clearly has a key role in transporting essential proteins into the prey and the TatA paralogues seem to play distinct roles in this.

Overall, our data clearly show that TatA1 is the most important TatA paralogue in *C. jejuni*, as deletion of the cognate gene resulted in severe effects on growth and the activities of many of the TAT substrate enzymes measured in this work. Nevertheless, although individual deletion of *tatA2* did not affect microaerobic growth (Fig. 2), that growth of the double mutant was more severely inhibited compared with the *tatA1* single mutant does suggest a role for TatA2 under respiratory conditions with oxygen as the electron acceptor. The TAT dependency of the Rieske iron–sulphur subunit of the cytochrome *bc*_1_ complex is the most likely reason for these growth defects (Bachmann *et al.*, 2006; Hitchcock *et al.*, 2010) and the data imply TatA1 has a dominant but not exclusive role in its assembly.

The position of the *tatA2* gene immediately downstream of the characterized *nap* gene cluster (Pittman *et al.*, 2007; Fig. 1) initially suggested a specific role in the assembly of the periplasmic nitrate reductase system. Indeed, RNaseq analysis of the NCTC 11168 transcriptome (Dugar *et al.*, 2013) has shown that *tatA2* is expressed from the primary *napA* promoter along with all of the *bona fide nap* genes. However, although a reduction in NapA-specific activity was found in the periplasm of the *tatA2* mutant (Fig. 3), this did not result in a noticeable growth defect under oxygen-limited conditions with nitrate as electron acceptor (Fig. 2). Our data thus suggest that *tatA2* is not specifically required for NapA translocation. We found three classes of TAT protein substrates that had differing dependency patterns on TatA1 and TatA2 based on the phenotypes of the *tatA1* and *tatA2* mutants. For several enzymes, including sulphite oxidase (SorA), the multicoper oxidase (CueO) and alkaline phosphatase (PhoX), complete dependency on TatA1 was observed as their activities in the *tatA2* mutant were identical to or greater than in wild-type cells, while they were abolished in the *tatA1* mutant. For nitrate reductase, formate dehydrogenase (FdhA) and TMAO reductase (TorA) a statistically significant reduction of specific activity in the *tatA2* mutant was seen, amounting to ~50 % for NapA and FdhA, suggesting that TatA2 could partially substitute for TatA1 (see Figs 4 and 5). Nevertheless, these activities were still abolished in intact cells of the *tatA1* mutant. This might be explained if there was some interaction between TatA1 and TatA2 such that a mixed complex was optimal for the translocation process. For TMAO reductase, the results were more complex as significant activity (~30 %) remained in the *tatA1* mutant cells and the reduction in activity in the *tatA2* mutant was less than that observed with NapA and FdhA. However, in the growth experiments with TMAO (Fig. 2), no growth was found with the *tatA1* mutant and a significant reduction was apparent with the *tatA2* deletion, supporting the involvement of both paralogues in TMAO reductase assembly.

The most interesting result bearing on the role of TatA2 was obtained in our studies on MfrA, the TAT signal peptide-containing flavoprotein active site subunit of the unusual periplasmic fumarate reductase in *C. jejuni*. The Mfr enzyme (Juhnke *et al.*, 2009) is restricted to a limited number of *Epsilonproteobacteria*, and is thought to allow the use of non-transportable fumarate analogues such as mesaconate and crotonate as electron acceptors as well as more rapid adaptation to fumarate respiration under low oxygen conditions (Guccione *et al.*, 2010). The specific rate of fumarate reduction catalysed by MfrA was similar in periplasmic fractions of both the *tatA1* and the *tatA2* mutants and only deletion of both genes abolished activity (Fig. 6), indicating redundancy of function of the TatA paralogues for the translocation of MfrA. Thus, despite being encoded in the *nap* operon, a major role for TatA2 could be in MfrA assembly. However, immunoblotting showed that unprocessed MfrA accumulated in the periplasm of the *tatA1* mutant while complementation restored the processing defect to normal. The data imply that the pre-protein form of MfrA can be translocated, without signal peptide cleavage, through TatA2 when this is the sole TatA paralogue in the cells. However, in the absence of TatA1, although TatA2 must be able to form transport-competent TatA2BC complexes, these complexes do not seem to be functionally equivalent to TatA1BC complexes and/or do not allow signal peptidase 1 to cleave the MfrA pre-protein. Examination of the MfrA signal peptide does not suggest any obvious differences, which might indicate a distinct translocation pathway, compared with other TAT substrates in *C jejuni*. Furthermore, the presence of the Mfr complex is not correlated with the presence of two TatA paralogues; the Mfr-containing *C. curvus*, *C. concisus* and *Wolinella succinogenes* have only one *tatA* gene (Table S1).

Together, our results suggest that in the absence of TatA1, TatA2 is not able to function correctly in the transport of any of the substrates tested. This contrasts with, for example, the *E. coli* TatE protein which can function independently in the absence of TatA. Significantly, TatA2 is lacking two functionally important residues: Phe39 (at the C terminus of the amphipathic helix) and Gln8 (at the N terminus of the transmembrane helix) (*E. coli* numbering; see Fig. 1). Phe39 appears to be absolutely conserved across TatAs, but in TatA2 is substituted (non-conservatively) with a glutamate. Position 8 in proteobacteria is conserved as a charged residue, but in TatA2 this is a phenylalanine. Both Gln8 and Phe39 have been shown to be required for TatA translocation function (Greene *et al.*, 2007; Hicks *et al.*, 2003) and are key to the latest membrane thinning model of TatA pore formation (Rodriguez *et al.*, 2013). In addition, TatA2 has a truncated N terminus, which might also have functional implications. Thus, it seems most likely that TatA2 would have to interact with TatA1 to form a fully functional complex. If there is interaction between TatA1 and TatA2, then analysis of single null mutants in each gene might not give a full picture of their roles and other methods will be necessary to determine the precise function of each of these proteins in translocation.

Cofactor insertion in TAT substrate enzymes in the cytoplasm is achieved by REMPs or TAT chaperones, although in some cases general cytoplasmic chaperones may additionally act on TAT substrates (Graubner *et al.*, 2007; Oresnik *et al.*, 2001). For example, DnaK is involved in TAT targeting of the *E. coli* multi-copper oxidase CueO (Graubner *et al.*, 2007) and SlyD is necessary for assembly of hydrogenases in *E. coli* (Zhang *et al.*, 2005), although we found this was not the case in *C. jejuni* (Howlett *et al.*, 2012). In fact in *C. jejuni*, only two obvious REMPs are encoded in the genome (Turner *et al.*, 2004), FdhM (Cj1514) and Cj0785. We previously demonstrated that, despite being a member of the TorD family, FdhM is not required for maturation of the TMAO reductase TorA (Cj0264), but is essential for the activity of formate dehydrogenase, consistent with the location of the cognate gene upstream of FdhA (Hitchcock *et al.*, 2010). Here, we found that the other *C. jejuni* REMP, a NapD homologue encoded by *cj0785* in an operonic arrangement with the periplasmic nitrate reductase (*nap*) structural genes (Fig. 1), was both essential and specific for nitrate reductase activity and nitrate-dependent oxygen-limited growth, as evidenced by the clear phenotypes of a *napD* deletion mutant and a *napD/fdhM* double mutant (Figs 7 and 8). The insertion mutant (*napD* : : *cat*) retained significant nitrate reductase activity and was able to grow comparably to the wild-type on nitrate under conditions of oxygen limitation (Fig. 7). The *C. jejuni* NapD (NapD_Cj_) has 112 amino acids compared with only 87 residues in the *E. coli* protein (NapD_Ec_). Protein secondary structure predictions (Jpred v3; Cole *et al.*, 2008) predicts that NapD_Cj1-74_ adopts the same ferredoxin-type fold seen in NapD_Ec_ (Maillard *et al*., 2007) but with an additional C-terminal sequence (amino acids 83–112), some of which may be structured. The *Swa*I restriction site used for insertion of the antibiotic resistance cassette in the *C. jejuni napD* gene fortuitously lies at the junction between where the similar *E. coli* ferredoxin-type fold ends and the extra *C. jejuni* C-terminal sequence begins, probably producing a truncated NapD protein. This might be unstable or produced in lower amounts, but sufficient for maturation of some NapA protein, resulting in the residual activity and growth observed in the insertion mutant. Attempts to corroborate this using an anti-NapD_Ec_ antibody (kindly provided by Frank Sargent, University of Dundee, UK) were unsuccessful due to lack of cross-reaction (data not shown). Given the evident specificity of NapD and FdhM, general cytoplasmic chaperones are probably involved in the translocation of other cofactor-containing TAT substrates in *C jejuni.*
